# Spiclomazine Induces Apoptosis Associated with the Suppression of Cell Viability, Migration and Invasion in Pancreatic Carcinoma Cells

**DOI:** 10.1371/journal.pone.0066362

**Published:** 2013-06-20

**Authors:** Wenjing Zhao, Dan Li, Zuojia Liu, Xiliang Zheng, Jin Wang, Erkang Wang

**Affiliations:** 1 State Key Laboratory of Electroanalytical Chemistry, Changchun Institute of Applied Chemistry, Chinese Academy of Sciences, Changchun, Jilin, China; 2 Department of Chemistry and Physics, State University of New York, Stony Brook, New York, United States of America; Bauer Research Foundation, United States of America

## Abstract

The effective treatment for pancreatic carcinoma remains critically needed. Herein, this current study showed that spiclomazine treatment caused a reduction in viability in pancreatic carcinoma cell lines CFPAC-1 and MIA PaCa-2 *in vitro*. It was notable in this regard that, compared with pancreatic carcinoma cells, normal human embryonic kidney (HEK-293) and liver (HL-7702) cells were more resistant to the antigrowth effect of spiclomazine. Biochemically, spiclomazine treatment regulated the expression of protein levels in the apoptosis related pathways. Consistent with this effect, spiclomazine reduced the mitochondria membrane potential, elevated reactive oxygen species, and activated caspase-3/9. In addition, a key finding from this study was that spiclomazine suppressed migration and invasion of cancer cells through down-regulation of MMP-2/9. Collectively, the proposed studies did shed light on the antiproliferation effect of spiclomazine on pancreatic carcinoma cell lines, and further clarified the mechanisms that spiclomazine induced apoptosis associated with the suppression of migration and invasion.

## Introduction

Pancreatic carcinoma is one of the most aggressively gastrointestinal malignancies with poor prognosis [1]–[3]. The current standard treatment for patients with pancreatic carcinoma is surgery, radiation and drugs, singly or in combination. However, the overwhelming majority of patients preclude surgery since they present at a locally advanced or metastatic stage. This means that pancreatic carcinoma has a relatively high mortality rate when compared with other cancers. The root of the problem lies in its insidious invasion, early metastasis, and resistance to conventional therapy [4], [5], which leads to the recurrence of tumor and the treatment failure and therefore presents an unfavorable prognosis. To address these problems, new therapeutic strategies for treatment of pancreatic carcinoma are imminently needed. Especially, it is of urgent importance for the development of effective anticancer drugs [3].

The understanding of the cellular and molecular mechanisms of pancreatic carcinoma is rapidly expanding and being hopefully utilized to develop better therapeutic drugs [6]. Since the inhibition of apoptosis plays central roles in the degradation process of cancers, apoptotic inducers as therapeutic drugs were widely used in trials [7]–[9]. Some works revealed that apoptosis-inducing drugs were with limited toxicity to normal cells and organs, apoptotic-mediating therapy therefore was an importance of direction for the therapy in cancers [10]–[12]. One has to keep in mind, however, that for some tumors multiple pathogeneses were observed [13]. Tumor metastases are the most common causes of death in cancer patients and the biggest challenges for cancer treatment. Cell migration plays a vital role in the progression of cancer since its deregulation causes the metastasis of tumors [14]. Also, cell invasion is an important characteristic of tumors in the process of metastasis. In addition, matrix metalloproteinases (MMPs) were believed to play a critical role in tumor invasion [15]. Thus, a better understanding of the underlying mechanisms of metastasis is important for the development of anticancer agent to improve the clinical outcome.

**Figure 1:**
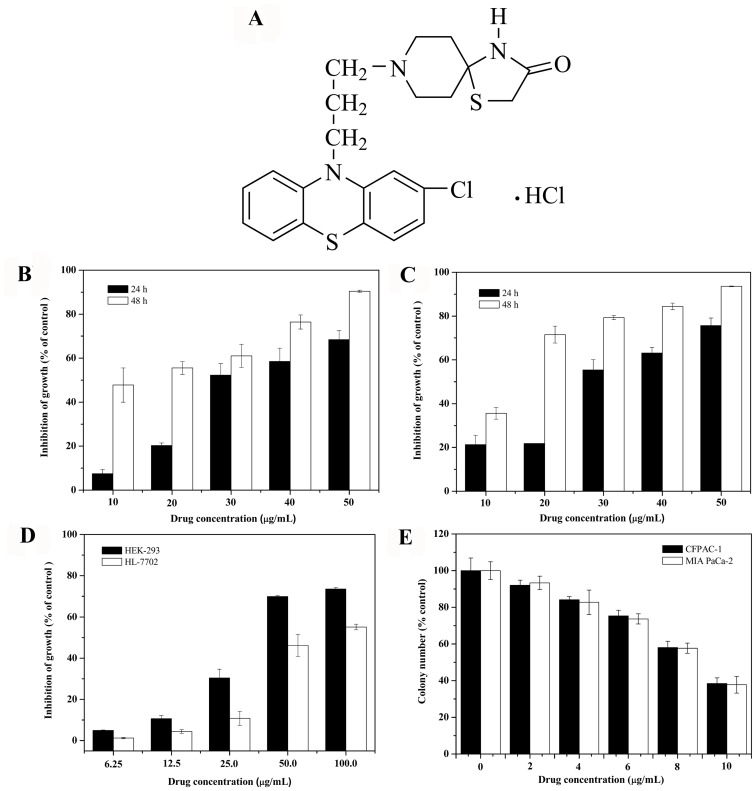
Spiclomazine reduces contact-dependent and -independent proliferation. (A) The molecular structure of spiclomazine. After cells were treated, the growth inhibition was assessed by MTT (B, C, D) and colony formation assays (E), respectively. Each value represents mean ± SD in three independent experiments.

Spiclomazine, termed as 1-Thia-4,8-diazaspiro[4.5]decan-3-one,8-[3-(2-chloro-10H -phenothiazin-10-yl)propyl]-hydrochloride (NSC290956, Figure 1A), demonstrated an antipsychotic effect as described in NCI Cancer Screen Current Data (http://dtp.nci.nih.gov). So far no studies have been reported on its effect on human pancreatic carcinoma. To search for a therapeutic agent, we used two pancreatic carcinoma cell lines CFPAC-1 and MIA PaCa-2 as model system to examine the anticancer property of spiclomazine. In accordance with our studies, spiclomazine effectively induced CFPAC-1 and MIA PaCa-2 cell death in the mitochondrial- mediated pathway *in vitro*, which promoted apoptosis while inhibiting viability, migration, and invasion. Of interest is the observation that the cytotoxic effect of spiclomazine on cancer cells was more pronounced than normal human embryonic kidney (HEK-293) and liver (HL-7702) cells. Overall speaking, we generated enough preliminary *in vitro* evidence to support the role of spiclomazine as an apoptotic inducer associated with the suppression of tumor metastasis. With further in-depth investigations, spiclomazine may be of value as a therapeutic agent aimed at combating pancreatic carcinoma progress and metastasis.

## Materials and Methods

### Materials

Media (DMEM and IMDM) and fetal bovine serum (FBS) were purchased from Gibico (Grand island, NY). All the antibodies used were purchased from BD Bioscience (San Jose, CA). Chemicals were purchased from Sigma (St Louis, MO). Spiclomazine was kindly supplied from NCI/DTP Open Chemical Repository (http://dtp.cancer.gov) and further confirmed by HPLC and MS. Spiclomazine was dissolved in DMSO to make stock solution (10 mg/mL) and further diluted to appropriate concentrations with double distilled water containing 10% DMSO immediately before use. The final concentration of DMSO in the culture media is 0.1%, which does not significantly affect on the cells.

### Cell Culture

Human pancreatic carcinoma cell lines CFPAC-1 and MIA PaCa-2 were purchased from American Type Culture Collection (ATCC, Rockville, MD) and maintained in IMDM and DMEM containing 10% FBS, 100 units/mL penicillin, 50 µg/mL streptomycin, and 100 µg/mL amphotericin (Invitrogen, Carlsbad, CA), respectively. Normal human embryonic kidney (HEK-293) and liver (HL-7702) cells were purchased from Chinese Academy of Science Type Culture Collection (Shanghai, China) and incubated in DMEM media containing 10% FBS. Cells were incubated in 25 mL flasks and kept in a humidified atmosphere with 5% CO_2_ at 37°C. During the process of cell culture, there was not any effect of mycoplasmas on these cell lines used, which was confirmed by a fluorochrome DNA staining test using a mycoplasma stain assay kit (Beyotime Institute of Biotechnology, China).

### MTT Assay

To measure cell viability, both human pancreatic carcinoma CFPAC-1 and MIA PaCa-2 and normal human HEK-293 and HL-7702 cells were seeded onto 96-well plate (1×10^4^ cells/well). After overnight incubation, the culture media was removed and treated by vehicle as control groups or different concentrations of spiclomazine in complete medium as experimental groups, respectively. After 24 and 48 h treatment, thiazolyl blue tetrazolium bromide (MTT) was added to each well and incubated for additional 4 h. The absorption of formazan solubilized in 100 µL of DMSO was measured at the wavelength of 490 nm by a 96-well multiscanner autoreader (Biotech Instruments, New York). MTT does not interfere with spiclomazine and causes a positive response.

### Colony Formation Assay

Pancreatic carcinoma cells (1×10^4^) were resuspended in 0.3% agar in DMEM or IMDM with 10% FBS and overlaid on 0.6% agar in the same media in 3.5 cm dishes. Cells were cultured with vehicle as control groups or various concentrations of spiclomazine as experimental groups at 37°C. The colonies fixed with 2.5% glutaraldehyde were evaluated by counting the colonies under Olympus X71 inverted phase microscope (Dr. Schumann Optik OHG, Hessen, Germany) after 10 days. Colony forming efficiency was calculated by the number of colonies/100 seeded cells.

### Hoechst 33342 Staining Assay

Both pancreatic carcinoma cells (1×10^5^) were respectively seeded onto glass- bottomed plate and incubated overnight. Thereafter, cells were treated by using either vehicle as control groups or various concentrations of spiclomazine as experimental groups, and then cultured for 48 h at the same conditions. Thereafter, cells were stained using Hoechst 33342 kit (KeyGEN Biotech, Nanjing, China) and observed using confocal-laser scanning microscope (TCS SP2, Heidelberg, Germany).

### Flow Cytometric Cell Apoptosis Detection

For quantitative evaluation of apoptosis, both pancreatic carcinoma cells (1×10^6^ cells/well in 24-well plate) after being treated by vehicle as control groups or various concentrations of spiclomazine as experimental groups were stained using an apoptosis detection kit (KeyGEN Biotech, Nanjing, China), and subsequently subjected to flow cytometry (FCM) using fluorescence activated cell sorter FACSAria (BD Bioscience, San Jose, CA). The apoptotic population was defined using Diva 6.0 software (BD Bioscience, San Jose, CA). In total, 10,000 events were analyzed in each sample.

### Western Blotting

For Western blotting analysis, both pancreatic carcinoma cells (1×10^6^) were seeded onto 10 cm plate. After treatment with indicated concentrations of spiclomazine and vehicle for 24 h, whole-cell proteins or mitochondrial fractions were isolated and measured. Equal amount of protein was separated using 12% SDS-PAGE using the Mini Protein System (Bio-Rad, Marnes-la-Coquette, France) and then transferred to the polyvinylidene difluoride (PVDF) membranes. The membranes, after being blocked with 3% BSA in TBST, were incubated with specific antibodies (1∶500 dilution) against caspase-3, 8, and 9, cytochrome *c*, Bcl-2, and Bax. Following washing with TBST, the membranes were incubated with peroxidase-conjugated goat antimouse or antirabbit secondary antibody (1∶1000 dilution), respectively. The specific proteins were scanned by the Vilber Lourmat Imaging and Gel Documentation System (Vilber Lourmat, France). Actin was used as internal positive control.

### Measurement of the Mitochondria Membrane Potential (ΔΨm)

Rhodamine-123 (Rho-123) dye was used to detect the changes in ΔΨm [16]. Cells (5×10^4^ cells/well) were cultured in 24-well plate at 5% CO_2_ and 37°C conditions. After a period of exposure (48 h) with vehicle or various concentrations spiclomazine, cells were incubated with Rho-123 (10 µg/mL) and subsequently subjected to FCM. In total, 10,000 events were analyzed in each sample.

### Detection of Reactive Oxygen Species (ROS)

Detection of ROS was performed by flow cytometric analysis as described previously [17]. In brief, cells (5×10^4^ cells/well) were cultured in 24-well plate at 5% CO_2_ and 37°C conditions. After 2 h exposure with vehicle or various concentrations spiclomazine, cells were subjected to FCM to determine ROS levels. In total, 10,000 events were analyzed in each sample.

### Wound-healing Assay

Cell migration was assessed by a wound-healing assay [18]. Pancreatic carcinoma cells grown to 90% confluence in 6-well plate (5×10^5^ cells/plate) were incubated for 24 h in starvation medium. Thereafter, a wound scratch was created by scratching the monolayer of cells with a pipette tip. Then, cells were washed three times with PBS and fresh culture media supplemented with 1% FBS was replaced. At *t* = 0, cells were treated with vehicle or 30.0 µg/mL spiclomazine. At *t* = 24 h, visualization of the distance of wound closure (compared with control at *t* = 0) was observed with an Olympus X71 inverted phase microscope.

### Invasion Assay

Fibroblast migration assay was performed using a 24-well transwell Boyden chamber (BD Biosciences). To prepare conditioned media, NIH3T3 mice fibroblast cells obtained from Chinese Academy of Science Type Culture Collection (Shanghai, China) were cultured in complete DMEM media to reach a confluent monolayer at 37°C in a humidified incubator with 5% CO_2_. Thereafter, cells were incubated in starvation media as above for 24 h. Then, culture supernatants were collected and sterilized by filtration. 10^5^ cells followed by incubation for 24 h and resuspended in starvation media were seeded in the upper compartment without coated by Matrigel (BD, San Jose, CA). While 0.5 mL mixed media (conditioned media : complete media = 1∶ 1) were filled in the lower compartment that served as a chemo-attractant. Cells were allowed to invade for 24 h before the Matrigel was removed, and invaded cells were fixed with methanol and stained with 1% crystal violet. Migration was quantified in three random fields in each well. Cells adhering to the bottom surface of the membrane were observed under microscopy.

### Gelatin Zymography

To investigate the gelatinase activity of MMPs, the gelatin zymography was performed. Both CFPAC-1 and MIA PaCa-2 cells cultured in serum-free IMDM or DMEM media were treated by spiclomazine for 24 h and then cell supernatants were collected. Gelatin zymography was carried out by subjecting conditioned media samples to 10% SDS-PAGE containing 1 mg/mL of gelatin. MMPs were activated by incubation at 37°C for 12 h in the activating buffer [19]. Gels were then stained with 0.25% Coomassie brilliant blue R-250 diluted in 40% methanol and 5% acetic acid. The transparent bands were scanned by using the Vilber Lourmat Imaging and Gel Documentation System (Vilber Lourmat, France).

### Statistical Analysis

Results were presented as means ± SD of at least triplicate experiments, each condition performed with three cultures. Statistical analyses were performed using SPSS 11.5 statistical software.

## Results

### Spiclomazine Reduces Contact-dependent and -independent Proliferation

Treatment with spiclomazine for 24 and 48 h resulted in a time- and dose-dependent growth reduction of both cell lines examined by MTT assay (Figure 1B, C). This small molecule inhibited the cell growth to approximately 61.1±5.3%, 76.4±3.2% and 90.4±0.5% of control in CFPAC-1 cells, and 79.3±1.0%, 84.5±1.5% and 93.6±0.2% of control in MIA PaCa-2 cells at the concentrations of 30, 40, and 50 µg/mL for 48 h, respectively. In comparison with the cytotoxic effect on pancreatic carcinoma cells, spiclomazine exhibited less cytotoxicity to normal HEK-293 and HL-7702 cells (Figure 1D). At the concentration of 50 µg/mL, spiclomazine inhibited cell growth to approximately 69.9±0.5% of the control in HEK-293 cells and 46.2±5.3% in HL-7702 cells after treatment for 48 h, respectively. The 50% inhibiting concentration (IC_50_) for 48 h treatment was 15.2±2.0 µg/mL (31.5±2.0 µM) for CFPAC-1, 12.9±0.9 µg/mL (26.8±0.9 µM) for MIA PaCa-2, 41.9±1.4 µg/mL (86.9±1.4 µM) for HEK-293, and 71.2±3.3 µg/mL (147.7±3.3 µM) for HL-7702, respectively. To determine long-term effect of spiclomazine, further, the ability of CFPAC-1 and MIA PaCa-2 cells to grow in an anchorage independent fashion was measured by colony formation assay. Our data indicated that spiclomazine inhibited contact-independent colony formation of both pancreatic carcinoma cells in a dose-dependent manner (Figure 1E). These results provide evidence that spiclomazine has selective cytotoxicity for both CFPAC-1 and MIA PaCa-2 cells.

### Induction of Apoptosis

**Figure 2:**
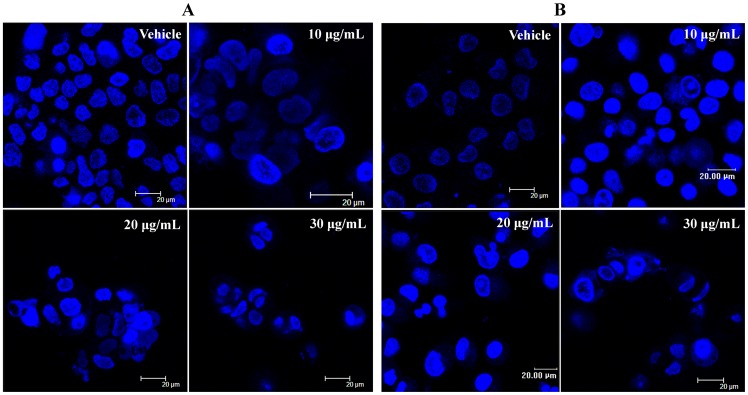
Spiclomazine induces morphological alterations. After cells were treated with various concentrations of spiclomazine for 48 h, cells were stained with Hoechst 33342 and examined using confocal laser scanning microscopy.

The effect of spiclomazine on cell toxicity can be assessed by measuring cellular morphology [20]. The nuclear condensation occurs when cells are in apoptotic stage, implying that the morphological changes are evident by nucleus staining [21]. Hoechst 33342 staining showed that the apoptotic morphological changes, including cell shrinkage, cytoplasmic condensation and the formation of apoptotic bodies, were observed after spiclomazine treatment (Figure 2).

**Figure 3:**
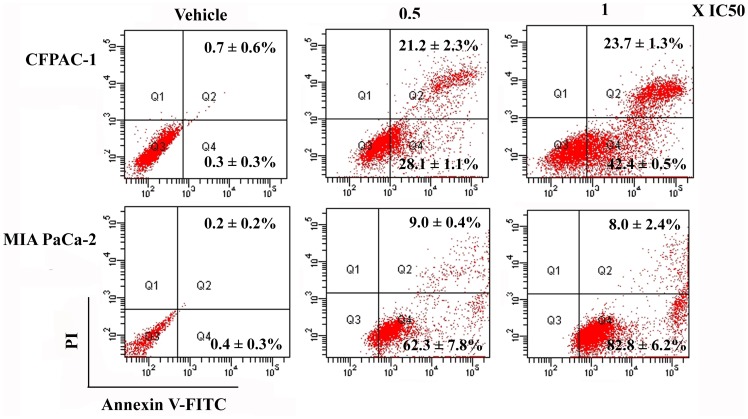
Spiclomazine induces apoptosis. Extent of apoptosis induced by spiclomazine was measured by FCM. Representative results from three independent experiments are shown; and results are presented as the mean ± SD of percent apoptotic cells from three independent experiments.

To investigate the cellular mechanisms underlying spiclomazine’s cytotoxic effect, we assessed the effect of spiclomazine on apoptosis in both cancer cells. Apoptotic cells were defined as the sum of Annexin V-FITC positive/PI negative (early apoptosis, Q4 quadrant) and Annexin V-FITC positive/PI positive (late apoptosis, Q2 quadrant) [22]. As shown in Figure 3, the number of early apoptotic cells increased from 0.3±0.3% in the control to 42.4±0.5% in the CFPAC-1 cells treated by IC_50_ concentration of spiclomazine. The number of early apoptotic cells increased from 0.4±0.3% in the control to 82.8±6.2% in the MIA PaCa-2 cells treated by IC_50_ concentration of spiclomazine. Taking the observations together, it is clear and accepted that the cytotoxic effect of spiclomazine on cancer cells is primarily mediated by inducing apoptosis.

### Effect of Spiclomazine on Proteins in the Apoptosis Related Pathways

**Figure 4:**
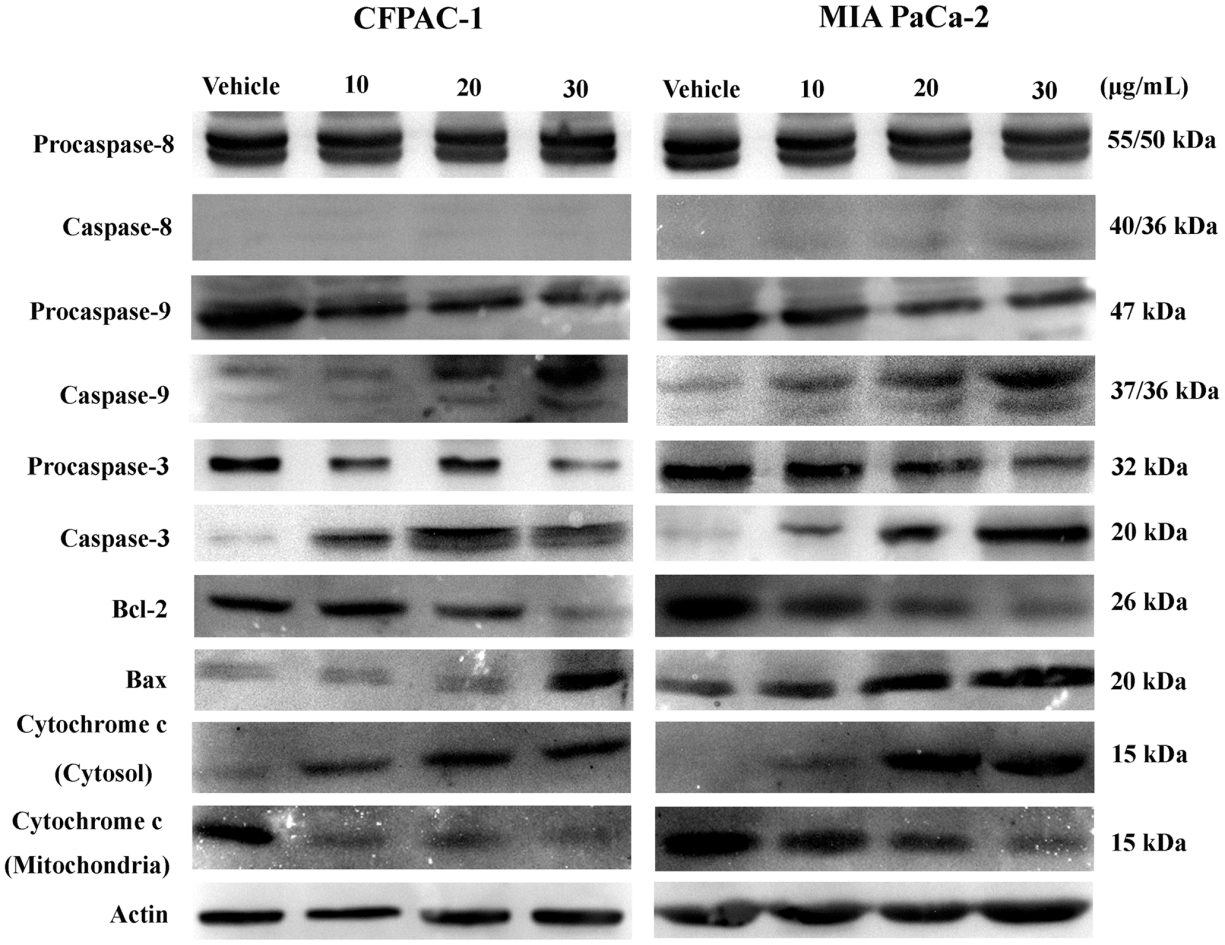
Western blotting of proteins. Equal amount of cellular proteins was separated by 12% SDS-PAGE gels and transferred to PVDF membranes. β-Actin was used to control loading.

The effect of spiclomazine on protein levels in the apoptosis related pathways was examined by western blotting assay (Figure 4). After being treated by spiclomazine, the cleavages of caspase-3/9 were increased in a dose-dependent manner. However, the cleaved-caspase-8 was almost undetectable in both cancer cells. In addition, the expression of Bax was up-regulated concomitant with the related attenuation of Bcl-2 protein expression. Simultaneously, the level of cytochrome *c* in cytosol was increased accompanied by the decrease of the level of cytochrome *c* in mitochondria.

### Spiclomazine Induces Apoptosis in the Mitochondrial Pathway

**Figure 5:**
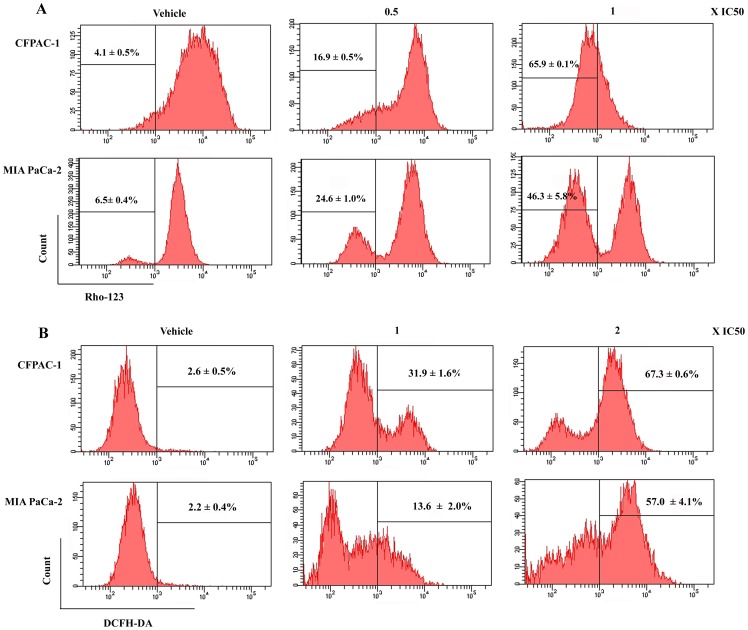
Analysis of the loss of ΔΨm (A) and the generation of ROS (B). The cells were treated at the indicated dose for 48 and 2 h and thereafter subject to FCM for the detection of ΔΨm and ROS, respectively. Representative results from three independent experiments are shown; and each value represents mean ± SD in three independent experiments.

A wide variety of drugs have been recognized to possess the ability to induce apoptosis in tumor cells via various mechanisms in the apoptotic pathways. On one hand, loss of ΔΨm is related to the mitochondrial apoptotic pathway [23]. As shown in Figure 5A, cells in control groups had high level ΔΨm, however, loss of ΔΨm was clearly observed in a dose-dependent manner in experimental groups. Experimentally, the loss of ΔΨm reached 16.9±0.5% and 65.9±0.1% when CFPAC-1 cells were treated with spiclomazine at 0.5 and 1×IC_50_ concentration, respectively. Also, the loss of ΔΨm collapse in MIA PaCa-2 cells reached 24.6±1.0% and 46.3±5.8% at the same concentrations, respectively. The data provided a support for the conclusion that pancreatic carcinoma cells treated by spiclomazine lost ΔΨm.

On the other hand, ROS generation is also linked to mitochondria [16]. As shown in Figure 5B, spiclomazine treatment significantly enhanced intracellular ROS level from 2.6±0.5% in the control to 67.3±0.6% in the treated CFPAC-1 cells at the concentration of IC_50_ for 2 h. Also, the ROS level was increased from 2.2±0.4% in the control to 57.0±4.1% in the treated MIA PaCa-2 cells at the same concentration.

### Spiclomazine Suppresses Cellular Motility *in vitro*

**Figure 6:**
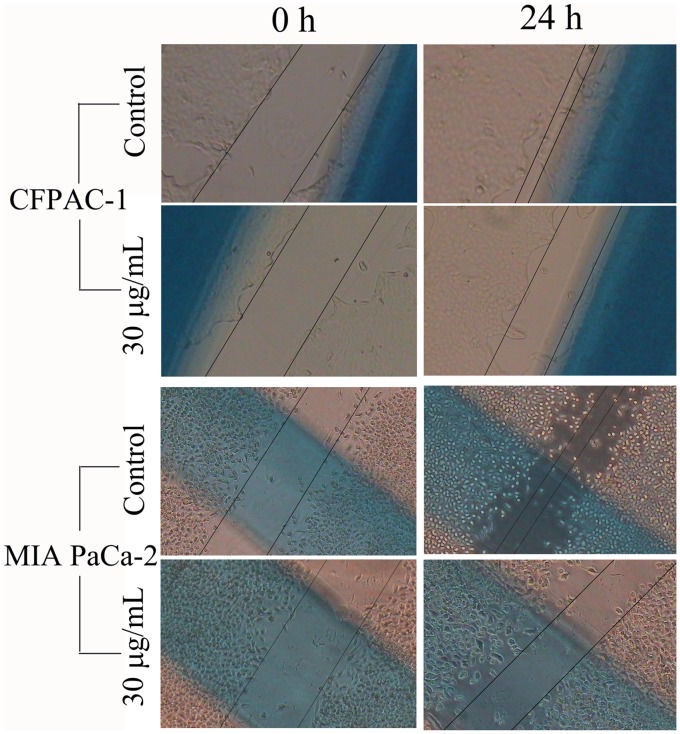
Spiclomazine suppressed cell migration determined by wound healing assay. The images of cell monolayer with scored wound at 0 h and 24 h after being incubated with 0.1%DMSO (final concentration) and 30.0 µg/mL spiclomazine were photographed by an inverted phase microscope. At *t* = 24 h, visualization of the distance of wound closure (compared with control at *t* = 0) was observed in three-independent wound sites per group.

It is well known that migration and invasion are the major characteristics of tumor metastases [18]. To examine whether spiclomazine has any effect on cellular motility, we first performed a wound-healing assay to test the cell migration upon spiclomazine treatment. After the addition of spiclomazine, the cells were allowed to migrate into the created cell-free area. As shown in Figure 6, in the absence of spiclomazine (control groups), both CFPAC-1 and MIA PaCa-2 cells migrated into the cell-free area. However, in the presence of spiclomazine at the concentration of 30 µg/mL (experimental groups), both cells were significantly inhibited to migrate into a wound to close the gap, which resulted in a much slower wound closure of the confluent monolayer.

**Figure 7:**
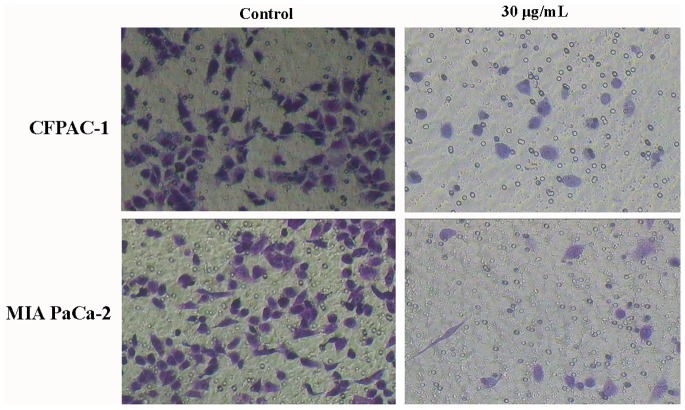
Spiclomazine inhibited cell invasion detected by transwell assay. Cells adhering to the bottom surface of the membrane were stained with 1% crystal violet and then observed under microscopy in three random fields in each well.

In order to evaluate the effect of spiclomazine on pancreatic carcinoma cells invasiveness, we next examined whether spiclomazine was able to suppress the invasion of both CFPAC-1 and MIA PaCa-2 cells through using Transwell chamber. As shown in Figure 7, spiclomazine markedly suppressed the migration of both pancreatic carcinoma cells in a dose-dependent manner. These results indicated that spiclomazine could suppress the mobility of both cancer cells *in vitro*.

**Figure 8:**
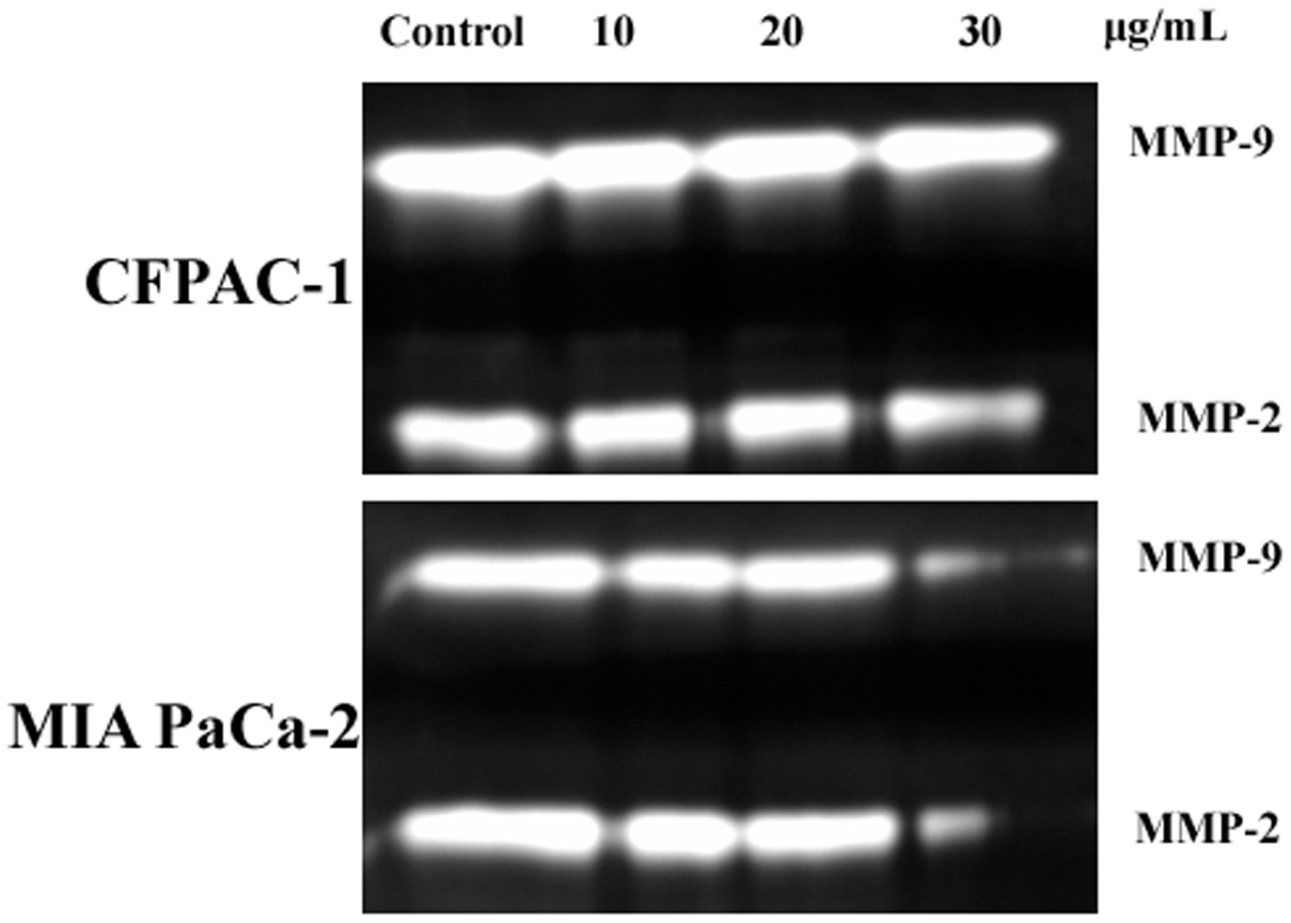
The gelatinase activity alterations of MMP-2/9 in both cells were assessed by a gelatin zymography assay. Gelatin zymography of both CFPAC-1 and MIA PaCa-2 cells was carried out by subjecting conditioned media samples to 10% SDS-PAGE containing 1 mg/mL of gelatin and then stained with 0.25% Coomassie brilliant blue R-250.

In the process of tumor invasion, MMPs play essential roles by degradating the basement membrane and collagenous extracellular matrix (ECM). To identify the possible alterations of MMPs in both cells, the activity of MMP-2/9 was assessed by a gelatin zymography. As shown in Figure 8, the intuitive activity changes of MMP-2/9 were observed in CFPAC-1 cells after being treated by spiclomazine at the concentration of 30 µg/mL. In addition, the significant low activities of MMP-2/9 were shown in MIA PaCa-2 cells at the concentration of 30 µg/mL. Although the activation degrees were different, the application of spiclomazine ultimately contributed to the activation of MMP-2/9. Collectively, our results suggested that spiclomazine exerted its effect on metastasis through the inhibition of migration and invasion in both CFPAC-1 and MIA PaCa-2 cells by down-regulating the expression of MMP-2/9.

## Discussion

In the present study, we demonstrated the occurrence and nature of spiclomazine effectively reducing pancreatic carcinoma cells activity (proliferation and migration). This is most likely attributable to the cytotoxic effect of spiclomazine on both pancreatic carcinoma cells. In contrast, spiclomazine showed a substantially reduced toxicity on normal cells including HEK-293 and HL-7702 at the same concentrations (Figure 1). In this regard, our results suggested that spiclomazine could exert cytotoxic effect selectively on pancreatic carcinoma cells. There are several possible explanations to account for this apparent lack of cellular toxicity. Possibly even more important is that normal cells have some protective mechanisms against spiclomazine by detoxifying excessive ROS, which is directly related to the reduction of the toxic effect of spiclomazine on HEK-293 and HL-7702 cells [24].

The apoptosis-inducing in tumor cells is considered very useful in the therapy of cancers [25]. Presently, a wide variety of compounds have been discovered to perform their pharmacological effects against some diseases through inducing apoptosis in various tumor cells of human origin [26]–[28]. As of now, some modes of action induced by therapeutic drugs in the apoptotic pathways have been delineated [29]–[34]. Herein, we were interested in testing the effect of spiclomazine on the apoptosis-inducing of CFPAC-1 and MIA PaCa-2 *in vitro*. Accumulating biochemical results indicated that spiclomazine treatment resulted in cleavage of pro-caspase-3/9 (Figure 4) indicating that spiclomazine induced apoptosis in pancreatic carcinoma cells. And these results prompted us to speculate that the intrinsic mitochondrial apoptotic pathway was activated [35].

Generally, cancer cells themselves are more prone to undergo apoptosis and a comprehensive understanding of the molecular pathways that regulate apoptosis in the intrinsic mitochondrial apoptotic pathway is important for developing new opportunities for the discovery of drugs [36]. To confirm the underlying apoptotic mechanisms, several proteins and molecular events related to apoptosis were examined. Release of cytochrome *c* from mitochondria was considered an apoptosis-specific characteristic in the process of apoptosis-inducing [37]–[39]. Bcl-2 involved in mediating apoptosis is an anti-apoptotic protein, and thus it acts as inhibitor of apoptosis through releasing cytochrome *c* from mitochondria and activating caspase-9 [40], [41]. Our findings that spiclomazine down-regulated the expression of Bcl-2 suggest that spiclomazine might induce both pancreatic carcinoma cells apoptosis. Bax, a member of Bcl-2 family, can promote cell death through regulating the mitochondrial apoptosis pathway [42]. The data shown in Figure 4 are compatible with the possibility that apoptosis was favored when increased levels of the pro-death Bax protein occurred. To further investigate the mechanism of apoptosis-inducing, we evaluated the effect of spiclomazine on ΔΨm. Loss of ΔΨm was observed as demonstrated in Figure 5A, which suggests that mitochondria is affected at the early apoptotic stage. Simultaneously, caspase-3 and -9 were activated as shown in western blotting (Figure 4), suggesting that the dissipation of ΔΨm played essential roles for the activation of the downstream effectors caspase-3 and -9 [43]. It is now clear that ROS production closely correlates with the potency inducing apoptosis by anti-cancer agents [44]. Spiclomazine elevated intracellular ROS levels as displayed in Figure 5B, which suggests that the enhancement of ROS levels following with the cleavage of caspases was sufficient for effective apoptosis-inducing in cancer cells [45]. These combined data clearly suggest that spiclomazine activated caspase-9 specifically in both cancer cells through the intrinsic mitochondrial pathway [46], which was mediated by the loss of ΔΨm and the generation of ROS.

Failure of treatment for pancreatic carcinoma is mainly caused by metastasis of tumor cells to the neighboring organs [14]. Migration and invasion are essential events in tumor metastasis. More compelling evidence for this possibility was given by the results summarized in Figure 6 and 7. We utilized the wound-healing assay to assess the motility of both CFPAC-1 and MIA PaCa-2 cells and the Transwell matrigel invasion assay to test the ability of both pancreatic carcinoma cells to penetrate ECM. The motility and invasion potential of CFPAC-1 and MIA PaCa-2 cells were strongly suppressed by a single application of spiclomazine. In the process of tumor invasion, the capacity of tumor cells to degradate the local matrix barriers is also required. MMPs can degradate the basement membranes and ECM, thus play pivotal roles in tumor invasion. MMP-2 and -9 are the principle MMPs expressed by cancer cells. As we did observe strong MMP-2 and -9 activity in both cancer cells in Figure 8, we speculate that MMP-2 and -9 are key players in metastasis of pancreatic carcinoma. Overall speaking, our studies showed a potential role of spiclomazine to suppress migration and invasion of highly metastatic pancreatic carcinoma cells *in vitro*. The exact molecular mechanisms by which spiclomazine suppresses pancreatic carcinoma metastasis remain to be awaited with interest.

In summary, spiclomazine results in decreased *in vitro* contact-dependent and -independent growth of pancreatic cells, coupled with activation of apoptotic cascades. Furthermore, spiclomazine demonstrates the inhibition of motility in pancreatic carcinoma cells *in vitro*, which is correlated with the suppression of migration and invasion. Taken together the appropriate results and mechanisms suggest that spiclomazine may be a promising option for treatment of patients with pancreatic carcinoma.

## References

[1] Jemal A, Siegel R, Ward E, Hao YP, Xu JQ, et al (2009) Cancer statistics 2009. CA Cancer J Clin 59: 225–249. 19474385 10.3322/caac.20006

[2] Jemal A, Siegel R, Xu J, Ward E (2010) Cancer statistics 2010. CA Cancer J Clin 60: 277–300. 20610543 10.3322/caac.20073

[3] Ducreux M, Boige V, Goere D, Deutsch E, Ezra P, et al (2007) Pancreatic cancer: From pathogenesis to cure. Best Practice & Reseach Clin Gastroenterol 21: 997–1014. 10.1016/j.bpg.2007.10.025 18070700

[4] Bardeesy N, DePinho RA (2002) Pancreatic cancer biology and genetics. Nat Rev Cancer 2: 897–909. 12459728 10.1038/nrc949

[5] Paul EO, Muhammad WS (2011) First-line treatment for advanced pancreatic cancer. JOP J Pancreas 12: 96–100. 21386629

[6] Stathis A, Moore J (2010) Advanced pancreatic carcinoma: current treatment and future challenges. Nat Rev Clin Oncol 7: 163–172. 20101258 10.1038/nrclinonc.2009.236

[7] Nicholson DW (2000) From bench to clinic with apoptosis-based therapeutic agents. Nature 407: 810–816. 11048733 10.1038/35037747

[8] Lu Y, Jiang F, Jiang H, Wu K, Zheng X, et al (2010) Gallic acid suppresses cell viability, proliferation, invasion and angiogenesis in human glioma cells. Eur J Pharmacol 641: 102–107. 20553913 10.1016/j.ejphar.2010.05.043 PMC3003697

[9] You BR, Park WH (2010) Gallic acid-induced lung cancer cell death is related to glutathione depletion as well as reactive oxygen species increase. Toxicol in Vitro 24: 1356–1362. 20417267 10.1016/j.tiv.2010.04.009

[10] Mimeault M, Hauke R, Batra SK (2007) Recent advances on the molecular mechanisms involved in the drug resistance of cancer cells and novel targeting therapies. Clin Pharmacol Ther 83: 673–691. 17786164 10.1038/sj.clpt.6100296 PMC2839198

[11] Murtaza I, Saleem M, Adhami VM, Hafeez BB, Mukhtar H (2009) Suppression of cFLIP by lupeol, a dietary triterpene, is sufficient to overcome resistance to TRAIL-mediated apoptosis in chemoresistant human pancreatic cancer cells. Cancer Res 69: 1156–1165. 19176377 10.1158/0008-5472.CAN-08-2917 PMC2996261

[12] David EF (1994) Apoptosis in cancer therapy: crossing the threshold. Cell 26: 539–542. 10.1016/0092-8674(94)90518-5 8069905

[13] Hanahan D, Weinberg RA (2000) The hallmarks of cancer. Cell 100: 57–70. 10647931 10.1016/s0092-8674(00)81683-9

[14] Keleg S, Büchler P, Ludwig R, Büchler MW, Friess H (2003) Invasion and metastasis in pancreatic cancer. Molecular Cancer 2: 14. 12605717 10.1186/1476-4598-2-14 PMC149416

[15] Chambers AF, Matrisian LM (1997) Changing views of the role of matrix metalloproteinases in metastasis. J Nat Cancer Inst 89: 1260–1270. 9293916 10.1093/jnci/89.17.1260

[16] Ren DD, Peng GH (2006) Effect of rhodoxanthin from potamogeton crispus L on cell apoptosis in Hela cells. Toxicol In Vitro 20: 1411–1418. 16919415 10.1016/j.tiv.2006.06.011

[17] Zou GM, Maitra A (2008) Small-molecule inhibitor of the AP endonuclease 1/REF-1 E3330 inhibits pancreatic cancer cell growth and migration. Mol Cancer Ther 7: 2012–2021. 18645011 10.1158/1535-7163.MCT-08-0113 PMC3569736

[18] Li Q, Wu M, Wang H, Xu G, Zhu T, et al (2008) Ezrin silencing by small hairpin RNA reverses metastatic behaviors of human breast cancer cells. Cancer Lett 261: 55–63. 18155831 10.1016/j.canlet.2007.11.018

[19] Chu CS, Xue B, Tu C, Feng ZH, Shi YH, et al (2007) NRAGE suppresses metastasis of melanoma and pancreatic cancer in vitro and in vivo. Cancer Lett 250: 268–275. 17140727 10.1016/j.canlet.2006.10.020

[20] Kerr JF, Wyllie AH, Currie AR (1972) Apoptosis: a basic biological phenomenon with wild-ranging implications in tissue kinetics. Br J Cancer 26: 239–257. 4561027 10.1038/bjc.1972.33 PMC2008650

[21] Beauparlant P, Shore GC (2003) Therapeutic activation of caspases in cancer: a question of selectivity. Curr Opin Drug Discov Dev 6: 179–187. 12669453

[22] Engeland MV, Nieland LJ, Ramaekers FC, Schutte B, Reutelingsperger CP (1998) Annexin V-affinity assay: a review on an apoptosis detection system based on phosphatidylserine exposure. Cytometry 31: 1–9. 9450519 10.1002/(sici)1097-0320(19980101)31:1<1::aid-cyto1>3.0.co;2-r

[23] Lisa BH, Cristina MP, Samuel C, Douglas RG (2008) Measuring apoptosis at the single cell level. Methods 44: 222–228. 18314052 10.1016/j.ymeth.2007.11.007 PMC2423010

[24] Evans DB, Abbruzzese JL, Rich TZ (1997) Cancer of the pancreas. In DeVita VT, Hellman S, Rosenberg SA, eds. Cancer, Principles and Practice of Oncology, Fifth Edition. Philadelphia: JB Lippincott Co. pp1054–1058.

[25] Desagher S, Martinou JC (2000) Mitochondria as the central control point of apoptosis. Trends in Cell Biol 10: 369–377. 10932094 10.1016/s0962-8924(00)01803-1

[26] Mutou Y, Ibuki Y, Terao Y, Kojima S Goto R (2008) Induction of apoptosis by UV-irradiated chlorinated bisphenol A in Jurkat cells. Toxicol in Vitro 22: 864–872. 18280695 10.1016/j.tiv.2008.01.001

[27] Pan MH, Huang YT, Chang CI, Ho CT, Pan BS (2007) Apoptotic-inducing epidioxysterols identified in hard clam (Meretrix lusoria). Food Chem 102: 788–795.

[28] Yarimar R, Juan R, Francisco A, Alfredo U, Mariugenia M, et al (2008) Cytotoxic and apoptosis-inducing effect of ent-15-oxo-kaur- 16-en-19-oic acid, a derivative of grandiflorolic acid from Espeletia schultzii. Phytochem 69: 432–438. 10.1016/j.phytochem.2007.07.025 17869315

[29] Ma D, Tremblay P, Mahngar K, Akbari-Asl P, Collins J, et al (2011) Induction of apoptosis and autophagy in human pancreatic cancer cells by a novel synthetic C-1 analogue of 7-deoxypancratistatin. Am J Biomed Sci 3: 278–291.

[30] Liu ZJ, Li D, Zhao WJ, Zheng XL, Wang J, et al (2012) A potent lead induces apoptosis in pancreatic cancer cells. PLoS ONE 7: e37841. 22745658 10.1371/journal.pone.0037841 PMC3380052

[31] Chen HM, Wu YC, Chia YC, Chang FR, Hsu HK, et al (2009) Gallic acid, a major component of *Toona sinensis* leaf extracts, contains a ROS-mediated anticancer activity in human prostate cancer cells. Cancer Lett 286: 161–171. 19589639 10.1016/j.canlet.2009.05.040

[32] Kurosu T, Ohki M, Wu N, Kagechika H, Miura O (2009) Sorafenib induces apoptosis specifically in cells expressing BCR/ABL by inhibiting its kinase activity to activate the intrinsic mitochondrial pathway. Cancer Res 69: 3927–3936. 19366808 10.1158/0008-5472.CAN-08-2978

[33] Sánchez AM, Malagarie-Cazenave S, Olea N, Vara D, Chiloeches A (2007) Apoptosis induced by capsaicin in prostate PC-3 cells involves ceramide accumulation, neutral sphingomyelinase, and JNK activation. Apoptosis 12: 2013–2024. 17828457 10.1007/s10495-007-0119-z

[34] Chodon D (2007) Inhibition of cell proliferation and induction of apoptosis by genistein in experimental hepatocellular carcinoma. Mol Cell Biochem 297: 73–80. 17006617 10.1007/s11010-006-9324-2

[35] Budihardjo I, Oliver H, Lutter M, Luo X, Wang X (1999) Biochemical pathways of caspase activation during apoptosis. Ann Rev Cell Dev Biol 15: 269–290. 10611963 10.1146/annurev.cellbio.15.1.269

[36] Rastogi RP, Sinha RRP (2009) Apoptosis: molecular mechanisms and pathogenicity. EXCLI J 8: 155–181.

[37] Liu X, Kim CN, Yang J, Jemmerson R, Wang X (1996) Induction of apoptotic program in cell-free extracts: Requirement for dATP and cytochrome *c* . Cell 86: 147–157. 8689682 10.1016/s0092-8674(00)80085-9

[38] Reed JC, Green DR (2002) Remodeling for demolition: Changes in mitochrondrial ultrastructure during apoptosis. Mol Cell 9: 1–3. 11804577 10.1016/s1097-2765(02)00437-9

[39] Zamzami N, Kroemer G (2001) The mitochondrion in apoptosis: How Pandora's box opens. Nat Rev Mol Cell Biol 2: 67–71. 11413468 10.1038/35048073

[40] Kim R, Emi M, Tanabe K (2006) Role of mitochondria as the gardens of cell death. Cancer Chemother Pharmacol 57: 545–553. 16175394 10.1007/s00280-005-0111-7

[41] Willis SN, Adams JM (2005) Life in the balance: How BH3-only proteins induce apoptosis. Curr Opin Cell Biol 17: 617–625. 16243507 10.1016/j.ceb.2005.10.001 PMC2930980

[42] Neuzil J, Wang XF, Dong LF, Low P, Ralph SJ (2006) Molecular mechanism of ‘mitocan’-induced apoptosis in cancer cells epitomizes the multiple roles of reactive oxygen species and Bcl-2 family proteins. Febs Lett 580: 5125–5129. 16979626 10.1016/j.febslet.2006.05.072

[43] Li D, Liu ZJ, Zhao WJ, Zheng XL, Wang J, et al (2013) A small-molecule induces apoptosis and suppress metastasis in pancreatic cancer cells. Eur J Pharm Sci 48: 658–667. 23313624 10.1016/j.ejps.2012.12.023

[44] McCollum AK, Teneyck CJ, Sauer BM, Toft DO, Erlichman C (2006) Up-regulation of heat shock protein 27 induces resistance to 17-allylamino- demethoxygel-danamycin through a glutathione-mediated mechanism. Cancer Res 66: 10967–10975. 17108135 10.1158/0008-5472.CAN-06-1629

[45] Zhou Y, Zhang SP, Liu CW, Cai YQ (2009) The protection of selenium on ROS mediated-apoptosis by mitochondria dysfunction in cadmium-induced LLC-PK1 cells. Toxicol in Vitro 23: 288–294. 19135140 10.1016/j.tiv.2008.12.009

[46] Wang J, Wu A, Xu YF, Liu JW, Qian XH (2009) M2-A induces apoptosis and G2-M arrest via inhibiting PI3K/Akt pathway in HL60 cells. Cancer Lett 283: 193–202. 19435648 10.1016/j.canlet.2009.03.039

